# Antileishmanial Activity of Aldonamides and *N*-Acyl-Diamine Derivatives

**DOI:** 10.1100/tsw.2008.100

**Published:** 2008-07-31

**Authors:** Elaine S. Coimbra, Camila G. Almeida, Wilson V. Júnior, Roberta C. N. Dos Reis, Ana C. F. De Almeida, Luana S. M. Forezi, Mauro V. De Almeida, Mireille Le Hyaric

**Affiliations:** ^1^ Departamento de Parasitologia, Microbiologia e Imunologia, Instituto de Ciências Biológicas, Universidade Federal de Juiz de Fora, Cidade Universitária, 36036-900, Juiz de Fora, MG, Brazil; ^2^ Departamento de Quíca, Instituto de Ciências Exatas, Universidade Federal de Juiz de Fora, Cidade Universitária, 36036-900, Juiz de Fora, MG, Brazil

**Keywords:** aldonamide, Leishmania

## Abstract

A number of lipophilic *N*-acyl-diamines and aldonamides have been synthesized and tested for their *in vitro* antiproliferative activity against *Leishmania amazonensis* and *L. chagasi*. Ribonamides, having one amino group, displayed good to moderate inhibition of parasite growth. The best result was obtained for compounds 10 and 15 with IC_50_ against *L. chagasi* below 5 μM.

## INTRODUCTION

Leishmaniasis is caused by a species belonging to the genus *Leishmania*, a protozoan parasite spread by the bite of infected phlebotomine sand flies. The disease currently affects about 350 million people in 88 countries around the world[1]. The three main clinical syndromes are cutaneous leishmaniasis, mucocutaneous leishmaniasis, and visceral leishmaniasis (also known as kala-azar), which is fatal if untreated. In the last years, cases of HIV and visceral leishmaniasis coinfection have also been reported in 35 countries[2].

The current chemotherapeutic treatment for human leishmaniasis relies on a few drugs, such as pentavalent antimonials (pentostam and glucantime), amphotericin B, and more recently miltefosine[3]. The treatment is limited by cost, difficulty of administration, variability of the efficacy, toxicity, and emergence of resistant strains[4,5,6]. There is, thus, an urgent need for the development of novel, nontoxic, potent, and effective new treatments for this worldwide health problem.

Among the number of compounds tested in the last years against *Leishmania*, several lipophilic diamine derivatives have been described[7,8,9,10]. Polyamines, such as putrescine, spermidine, and spermine, are essential for cellular growth and proliferation in all living organisms. The antileishmanial activity of lipophilic diamines would be due to their interference with the polyamine metabolism pathway of the protozoa, different from the mammalian cells polyamine pathway[11,12,13]. The lipophilic part of the molecule facilitates its interaction with membrane lipids, allowing its penetration into the cytoplasm where it can act[14,15].

In this work, we report the synthesis and antileishmanial activity of two types of aldonamides derived from lipophilic diamines and *N*-acylated diamines (Fig. 1).

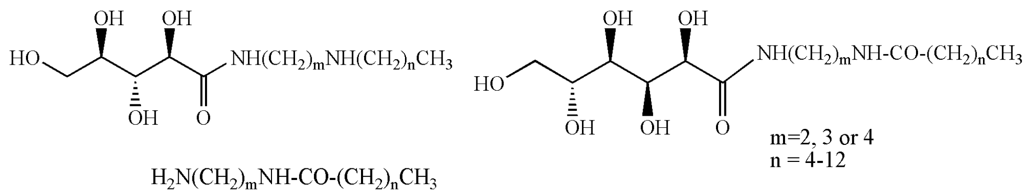

**FIGURE 1**. General structure of aldonamides and *N*-acylated diamines.

## MATERIAL AND METHODS

### Chemicals

Preparation of aldonamides **9–16** was achieved by the nucleophilic addition of *N*-alkylamines **1–8** to D-ribono-1,4-lactone (Scheme 1)[9,16,17]. *N*-Acylated diamines **22–31** were prepared by reaction of fatty esters **17–21** with either 1,2-ethanediamine or 1,4-butanediamine[18,19]. Reaction of the resultant amides with D-glucono-1,5-lactone led to aldonamides **32–41** (Scheme 1)[20].

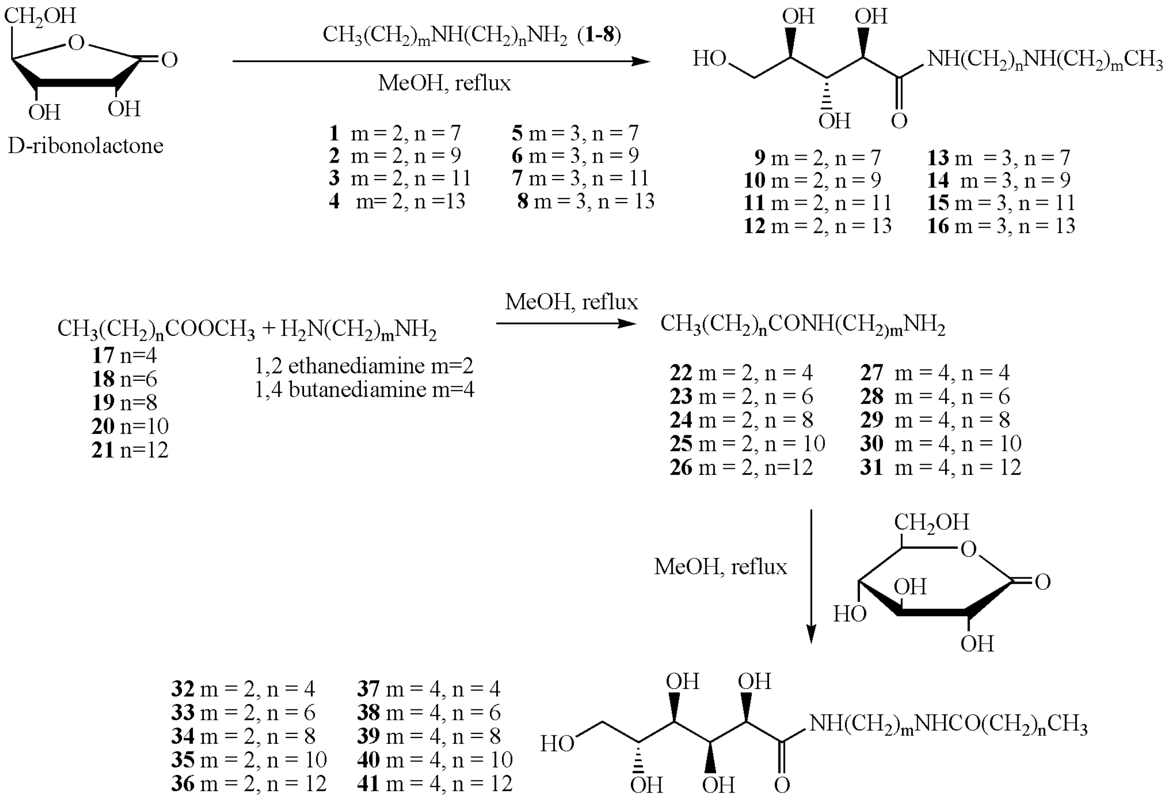

**SCHEME 1.** Synthesis of potential antileishmanial compounds.

### *In vitro* Evaluation

The antiproliferative activity of compounds **9–16** and **22–41** against *L. amazonensis* and *L. chagasi* was determined *in vitro* by the colorimetric MTT method based on tetrazolium salt reduction by mitochondrial dehydrogenases, as previously described[3,9]. The results are expressed as the concentrations inhibiting parasite growth by 50% ($IC_{50}$) after a 3-day incubation period. The $IC_{50}$ values represent means of three separate experiments. Amphotericin B was used as the reference drug and $IC_{50}$ values were of 0.9 and 1.9 μ*M* on *L. amazonensis* and *L. chagasi* promastigote forms, respectively.

## RESULTS AND DISCUSSION

The results are presented in Table 1. All ribonamides **9–16** displayed good to moderate activity against the two species of *Leishmania*. Compounds **10** and **15** were the most active against *L. chagasi* ($IC_{50}$ = 2.49 μ*M*) and *L. amazonensis* ($IC_{50}$ =11.8 μ*M*), respectively. Interestingly, in a general biological evaluation, the compounds tested were more active against the promastigote form of *L. chagasi*, the causal agent of fatal visceral leishmaniasis. Hydrosolubility seems to be important, as glycosylated compounds **9–16**, having a polar moiety, are more active than amides **22–31**. However, the antileishmanial activity of the ribonamides remains lower than that of the corresponding diamines **1–8**[9].

None of the tested gluconamides exibited antiproliferative activity. These compounds possess two amide groups in their structure, while in compounds **9–16,** only one of the nitrogen atoms is involved in an amide linkage. This suggests that the presence of at least one amine group is of great importance in the mechanism of action of these compounds against *Leishmania*.

In the *N*-acyl-diamine series, compound **30**, having a 12-carbon chain, was the only *N*-acylated butanediamine derivative showing activity. These compounds appeared to be less soluble than their ethylenediamine analogues, complicating the tests. Short-chain compounds **22**, **23**, **27**, and **28** were inactive. The best results were obtained for compounds **26** and **30**, which displayed moderated antiproliferative activity against the two species of *Leishmania*.

## CONCLUSION

In this work, we described the preparation and antileishmanial evaluation of three series of amides derived from lipophilic diamines. The best results were obtained with ribonamides **10** and **15**, showing that the presence of a polar group, enhancing the hydrosolubility of the compound, may be important. The results also showed that the presence of at least one amine group is necessary for the antiproliferative activity.

## ACKNOWLEDGMENTS

The authors gratefully acknowledge UFJF, CAPES, and CNPq for fellowships. This research was supported by FAPEMIG.

**TABLE 1**
***In vitro* Antiproliferative Activity against *L. amazonensis* and *L. chagasi* Promastigote Forms**

| Compound | *L. amazonensis* $IC_{50}$ (μ*M*) | *L. chagasi* $IC_{50}$ (μ*M*) |
|---|---|---|
| 9 | 41.98 (±2.40) | 4.80 (±0.60) |
| 10 | 35.00 (±9.20) | 2.49 (±0.79) |
| 11 | 47.40 (±6.70) | 19.0 (±0.70) |
| 12 | 40.48 (±0.35) | 11.45 (±1.31) |
| 13 | 40.49 (±2.40) | 31.28 (±1.99) |
| 14 | 20.57 (±2.66) | 11.65 (±1.67) |
| 15 | 11.80 (±0.30) | 4.33 (±0.20) |
| 16 | 19.60 (±0.23) | 8.10 (±0.86) |
| 22 | >227 | >227 |
| 23 | >227 | >227 |
| 24 | >227 | 16.67 (±0.98) |
| 25 | 26.70 (±0.05) | 10.10 (±0.04) |
| 26 | 19.03 (±0.05) | 9.94 (±0.29) |
| 27 | >227 | >227 |
| 28 | >227 | >227 |
| 29 | n.s | n.s |
| 30 | 21.55 (±0.32) | 8.48 (±0.16) |
| 31 | n.s | n.s |
| 32 | >227 | >227 |
| 33 | >227 | >227 |
| 34 | >227 | >227 |
| 35 | >227 | >227 |
| 36 | >227 | >227 |
| 37 | >227 | >227 |
| 38 | >227 | >227 |
| 39 | >227 | >227 |
| 40 | >227 | >227 |
| 41 | >227 | >227 |
| Amphotericin B | 0.90 (±0.07) | 1.90 (±0.25) |

## References

1. WHO (2005) Tropical Disease Research. Progress 2003–2004. Special Programme for Research and Training in Tropical Diseases. Geneva.
2. Moreno, J., Canavate, C., Chamizo, C., Laguna, F., and Alvar, J. (2000) HIV–Leishmania infantum co-infection: humoral and cellular immune responses to the parasite after chemotherapy. *Trans. R. Soc. Trop. Med. Hyg.* **94,** 328–332.
3. Mishra, J., Saxena, A., and Singh, S. (2007) Chemotherapy of leishmaniasis: past, present and future. *Curr. Med. Chem.* **14,** 1153–1169.
4. Ouellette, M., Drummelsmith, J., and Papadopoulou, B. (2004) Leishmaniasis: drugs in the clinic, resistance and new developments. *Drug Resist. Updat.* **7,** 257–266.
5. Croft, S.L., Barret, M.P., and Urbina, J.A. (2005) Chemotherapy and trypanosomiases and leishmaniasis. *Trends Parasitol.* **21,** 508–512.
6. Croft, S.L. and Coombs, G.H. (2003) Leishmaniasis--current chemotherapy and recent advances in the search for novel drugs. *Trends Parasitol.* **19,** 502–508.

7. Del Olmo, E.M., Alves, M., Lopéz, J.L., Inchaustti, A., Yaluff, G., and De Arias, A.R. (2002) Leishmanicidal activity of some aliphatic diamines and amino-alcohols. *Bioorg. Med. Chem. Lett.* **12,** 659–662.
8. Labadie, G.R., Choi, S.R., and Avery, M.A. (2004) Diamine derivatives with antiparasitic activities. *Bioorg. Med. Chem. Lett.* **14,** 615–619.
9. da Costa, C.F., Coimbra, E.S., Braga, G.B., Dos Reis, R.C.N., da Silva, A.D., and de Almeida, M.V. (2008) Preparation and antileishmanial activity of lipophilic *N*-alkyl diamines. *Biomed Pharmacother.* [Epub ahead of print]
10. Rebollo, O., del Olmo, E., Ruiz, G., López-Pérez, J.L., Giménez, A., and San Feliciano, A. (2008) Leishmanicidal and trypanocidal activities of 2-aminocyclohexanol and 1,2-cyclohexanediamine derivatives. *Bioorg. Med. Chem. Lett.* **18,** 184–187.
11. Muller, S., Coombs, G.H., and Walter, R.D. (2001) Targeting polyamines of parasitic protozoa in chemotherapy. *Trends Parasitol.* **17**, 242–249.
12. Bacchi, C.J. and Yarlett, N. (2002) Polyamine metabolism as chemotherapeutic target in protozoan parasites. *Mini Rev. Med. Chem.* **2,** 553–563.
13. Heby, O., Roberts, S.C., and Ullman, B. (2003) Polyamine biosynthetic enzymes as drug targets in parasitic protozoa. *Biochem. Soc. Trans.* **31,** 415–419.
14. Basselin, M. and Robert-Gero, M. (1998) Alterations in membrane fluidity, lipid metabolism, mitochondrial activity, and lipophosphoglycan expression in pentamidine-resistant *Leishmania Parasitol. Res.* **84,** 78–83.
15. Herrmann, H.O. and Gercken, G. (1982) Metabolism of 1-0-[1'-14C]octadecyl-sn-glycerol in Leishmania donovani promastigotes. Ether lipid synthesis and degradation of the ether bond. *Mol. Biochem. Parasitol.* **5,** 65–76.
16. Reis, R.C.N., Oda, S.C., De Almeida, M.V., Lourenço, M.C.S., Vicente, F.R.C., Barbosa, N.R., Trevizani, R., Santos, P.L.C., and Le Hyaric, M. (2008) Synthesis and antimicrobial activity of amphiphilic carbohydrate derivatives. *J. Braz. Chem. Soc.,* in press.
17. De Almeida, M.V., Saraiva, M.F., de Souza, M.V.N., da Costa, C.F., Vicente, F.R.C., and Lourenço, M.C.S. (2007) Synthesis and antitubercular activity of lipophilic moxifloxacin and gatifloxacin derivatives. *Bioorg. Med. Chem. Lett.* **17,** 5661–5664
18. Hill, A.J. and Aspinall, S.R. (1939) The preparation and cyclization of monoacylethylenediamines. *J. Am. Chem. Soc.* **61,** 822–825.
19. General procedure for the preparation of *N*-acyl diamines **22**–**31**. To a solution of 1,2-ethanediamine or 1,4-butanediamine (100 mmol) in 50 ml of ethanol at reflux was slowly added the fatty ester (20 mmol) in ethanol. The reaction mixture was maintained under reflux for 48 h. The solvent was then evaporated under reduced pressure and the residue was purified by recrystalization or column chromatography on silica gel (eluent: dichloromethane:methanol) furnishing compounds **22**–**31** in 60–90% yield.
20. General procedure for the preparation of aldonamides **32**–**41**. To a solution of D-gluconolactone (1 mmol) dissolved in 20 ml of hot methanol were added *N*-acylated diamine **22**–**31** (1 mmol). The reaction mixture was maintained under reflux for 72 h. The solvent was then evaporated under reduced pressure and the residue was purified by recrystalization, furnishing compounds **32**–**41** in 70–90%.
